# A comprehensive assessment of climate and topographic changes to identify conservation gaps for the critically endangered species *Coptis teeta* Wall in southwest China

**DOI:** 10.3389/fpls.2026.1901185

**Published:** 2026-08-31

**Authors:** Ran Li, Xiaobo Wang, Wende Chen, Yuelin Wang, Yigui Zhou, Jiali Zhou, Kening Xu, Xuan Qi, Zhongxuan Huang

**Affiliations:** 1 College of Geography and Planning, Chengdu University of Technology, Chengdu, China; 2 Sichuan Forestry and Grassland Survey and Planning Institute, Chengdu, China

**Keywords:** climate change, Coptis teeta Wall, endangered species, MAXENT model, potential habitat

## Abstract

**Introduction:**

*Coptis teeta* Wall, a nationally protected Grade II plant species endemic to China, is mainly distributed in the Yunnan and Sichuan provinces. Predicting its potential suitable habitats is essential for understanding its ecological responses to climate change and for supporting effective conservation planning.

**Methods:**

Based on 20 occurrence records of *Coptis teeta* Wall, this study employed bioclimatic and topographic variables derived from WorldClim to simulate habitat suitability under past, present, and future climate conditions using the MaxEnt model. Four future climate scenarios across two time periods were considered to assess potential distribution dynamics.

**Results:**

The results indicate that precipitation of the warmest quarter (Bio18) is the primary climatic factor governing the species’ distribution, while topographic variables such as elevation and slope contribute to habitat stability. Spatiotemporal analysis reveals that the suitable habitat area of *Coptis teeta* Wall exhibited a “decrease–increase” trend from the Last Glacial Maximum to the present, reaching its maximum extent under current climatic conditions. However, future projections under Shared Socioeconomic Pathway (SSP) scenarios suggest a general contraction of suitable habitats. Notably, the species is projected to shift not only toward higher elevations but also toward southeastern coastal regions of China. Under high-emission scenarios such as SSP370, intensified climate variability and winter warming (Bio6) are expected to exacerbate habitat fragmentation, substantially increasing extinction risk.

**Conclusion:**

Accordingly, Conservation priorities should include the existing core range in the Sichuan-Guizhou-Yunnan region, as well as potential future climate refuges identified along the Guizhou-Hunan border.

## 1 Introduction

Climate change profoundly influences plant habitat suitability, with underlying mechanisms generally classified into three main categories. First, global temperature change drives shifts in species distribution ranges by altering thermal environments. A European study reported that more than 100 plant species exhibited an average upslope shift of 29m per decade since the 20th century in response to climate warming (Lenoir et al., 2008). However, species migration rates often lag behind the pace of climate warming (Aitken et al., 2008), increasing the risk of habitat mismatch and population decline. Second, changes in global precipitation regimes constrain plant distributions, with rainfall patterns and soil moisture emerging as key limiting factors (Wang et al., 2016). Reduced precipitation can fragment suitable habitats, ultimately leading to habitat loss and population isolation, while soil water-holding capacity partly determines plant water-use efficiency (Kougioumoutzis et al., 2024). Finally, extreme climatic events affect plant habitat suitability primarily by disrupting phenological processes and increasing climate-related disturbances. Phenological disruption reduces reproductive success, whereas meteorological extremes such as heatwaves and cold spells substantially increase plant mortality (Hamann et al., 2021).

*Coptis teeta* Wall is a perennial herb belonging to the family Ranunculaceae and the genus Coptis, reaching 15–30 cm in height. Its rhizome is yellow and hook-shaped, bearing dense fibrous roots at the nodes. The species typically inhabits high-altitude regions at elevations ranging from 1,500 to 3,000 m. It possesses significant medicinal value and is primarily distributed in northwestern Yunnan, Sichuan Province, and southeastern Tibet, China (Chi et al., 2024; Liu et al., 2021). *Coptis teeta* Wall is listed as a Class II nationally protected plant in China and is categorized as Critically Endangered on the IUCN Red List of Threatened Species. Existing studies have mainly focused on phenotypic diversity and general conservation efforts (Huang et al., 2005; Yang et al., 2013). In contrast, research on habitat suitability prediction remains limited. Although habitat suitability studies have been conducted for endangered plants in Southwest China and for the genus Coptis (Liu et al., 2025, 2024; Xu et al., 2025), research specifically targeting *Coptis teeta* Wall remains insufficient. Predicting the potential suitable habitat of *Coptis teeta* Wall enables assessment of its future distribution patterns under climate change, thereby improving conservation effectiveness and providing a scientific basis for *ex situ* conservation and experimental cultivation (Dai et al., 2022).

Regarding species distribution prediction approaches, three major modeling frameworks have emerged in recent years. The first category comprises statistical models, including Generalized Linear Models (GLM), Generalized Additive Models (GAM), and Multivariate Adaptive Regression Splines (MARS) (Wang et al., 2025; Wang and Ni, 2006; Xia et al., 2020), which establish mathematical relationships between species occurrence and environmental variables based on statistical theory to generate predictive outcomes. The second category consists of machine learning models, such as Maximum Entropy (MaxEnt) (Bo et al., 2024; Wang et al., 2023; Xi et al., 2018), Random Forest (RF) (Breiman, 2001; Luo et al., 2025; Su et al., 2018), and Support Vector Machines (SVM) (González-Camacho et al., 2018; Montoya-Jimenez et al., 2022), which rely on automated learning algorithms to capture complex relationships between species distributions and environmental factors. The third category includes ecological niche models, such as BioCLIM, GARP (Genetic Algorithm for Rule-set Prediction), and CLIMEX (Rocchini et al., 2015; Wang et al., 2011), which project species distributions based on ecological niche theory. Among machine learning approaches, MaxEnt is the most widely applied model, as it estimates species probability distributions by maximizing entropy under environmental constraints, enabling reliable niche predictions even when occurrence data are limited (Virgili, 2018). Moreover, MaxEnt has been demonstrated to perform effectively with extremely sparse species distribution records (Zhang et al., 2024), making it particularly suitable for predicting the potential distributions of endangered species.

## 2 Materials and methods

### 2.1 Species data acquisition

Species occurrence data for *Coptis teeta* Wall were collected from the Digital Herbarium of China (CVH)(https://www.cvh.ac.cn/), the National Specimen Information Infrastructure (NSII)(http://www.nsii.org.cn/2017/homeMobile.php), and the Global Biodiversity Information Facility (GBIF)(https://www.gbif.org/). The latitude and longitude coordinates were compiled, standardized, and corrected to ensure data accuracy. Using SPSS, duplicate and erroneous records were removed, resulting in 20 valid occurrence points for *Coptis teeta* Wall used in subsequent analyses (**Table 1**).

**Table 1:** Geographical distribution of *Coptis teeta* Wall.

Serial number	Species	Longitude	Latitude	Data source
1	*Coptis teeta* Wall	110.040007	25.742153	GBIF
2	*Coptis teeta* Wall	113.184435	24.813781	GBIF
3	*Coptis teeta* Wall	98.869207	26.901731	GBIF
4	*Coptis teeta* Wall	100.10444	25.89444	GBIF
5	*Coptis teeta* Wall	102.74055	25.13638	GBIF
6	*Coptis teeta* Wall	103.086573	22.792186	GBIF
7	*Coptis teeta* Wall	103.349998	29.533333	GBIF
8	*Coptis teeta* Wall	106.633387	24.392902	GBIF
9	*Coptis teeta* Wall	107.099276	29.157849	GBIF
10	*Coptis teeta* Wall	107.26667	29.116667	GBIF
11	*Coptis teeta* Wall	109.213028	23.695857	GBIF
12	*Coptis teeta* Wall	111.009947	25.897942	GBIF
13	*Coptis teeta* Wall	113.341527	23.127041	GBIF
14	*Coptis teeta* Wall	96.344847	29.01669	GBIF
15	*Coptis teeta* Wall	98.568611	27.801389	GBIF
16	*Coptis teeta* Wall	98.665833	25.991667	GBIF
17	*Coptis teeta* Wall	98.784444	27.163056	GBIF
18	*Coptis teeta* Wall	98.857727	25.841921	GBIF
19	*Coptis teeta* Wall	99.16401	27.19361	CHV
20	*Coptis teeta* Wall	99.17694	26.62238	NSII

**Figure 1:**
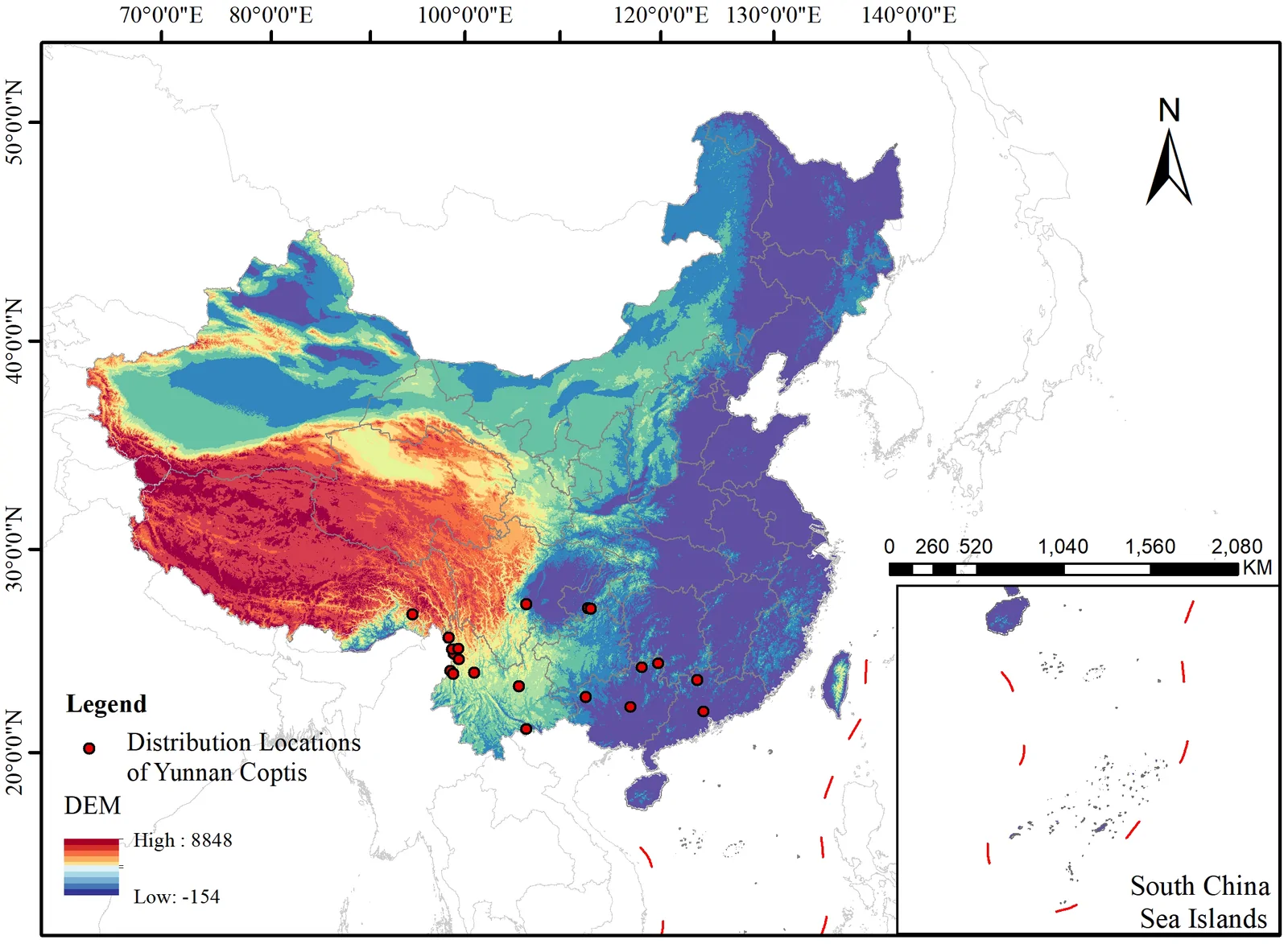
Geographic distribution of *Coptis teeta* Wall in China.

Digital Elevation Model (DEM) data for China were retrieved from the Geospatial Data Cloud (https://www.gscloud.cn/), while national administrative boundary data were obtained from the DataV data visualization platform (http://datav.aliyun.com/portal/school/atlas/area_selector). The occurrence records of *Coptis teeta* Wall were imported into ArcGIS 10.8 to visualize its current geographic distribution (**Figure 1**).

### 2.2 Environmental factor data acquisition

Bioclimatic data were retrieved from the WorldClim global climate database (https://worldclim.org/). These datasets encompassed elevation and climatic variables for several distinct periods, including the Last Glacial Maximum (LGM, ~26–20 ka BP), themid-Holocene (~8.5–4 ka BP), contemporary conditions, and future climate scenarios for 2041–2060 and 2061–2080. A total of twenty-three environmental variables were utilized at a spatial resolution of 2.5 arc-minutes (**Table 2**). The BCC-CSM2-MR (Beijing Climate Center Climate System Model) was selected to provide future climate projections for *Coptis teeta* Wall. To cover a comprehensive emission gradient, four Shared Socioeconomic Pathways (SSPs)—SSP126, SSP245, SSP370, and SSP585—were employed, representing low, medium, medium-to-high, and high carbon emission scenarios, respectively. In addition, soil pH data were acquired from the SoilGrids global database (https://soilgrids.org/).

**Table 2:** Climate environmental variable selection.

Code	Environmental variable	Unit
Bio1	Annual average temperature	°C
Bio2	Average daily temperature variation	°C
Bio3	Isotherm	%
Bio4	Seasonal Temperature Variation	–
Bio5	Highest Temperature in Hottest Month	°C
Bio6	Lowest temperature in the coldest month	°C
Bio7	Annual temperature range	°C
Bio8	Average Temperature of the Wettest Season	°C
Bio9	Average temperature of the driest season	°C
Bio10	Average temperature of the warmest season	°C
Bio11	Average temperature of the coldest season	°C
Bio12	Annual average precipitation	mm
Bio13	Precipitation in the wettest month	mm
Bio14	Precipitation in the driest month	mm
Bio15	Precipitation seasonality	–
Bio16	Precipitation in the wettest season	mm
Bio17	Precipitation during the driest season	mm
Bio18	Precipitation during the warmest season	mm
Bio19	Precipitation during the coldest season	mm
Elevation	Elevation	m
Aspect	Slope direction	°
Slope	Slope	%
Soil_PH	Soil pH	–

### 2.3 Data processing

**Figure 2:**
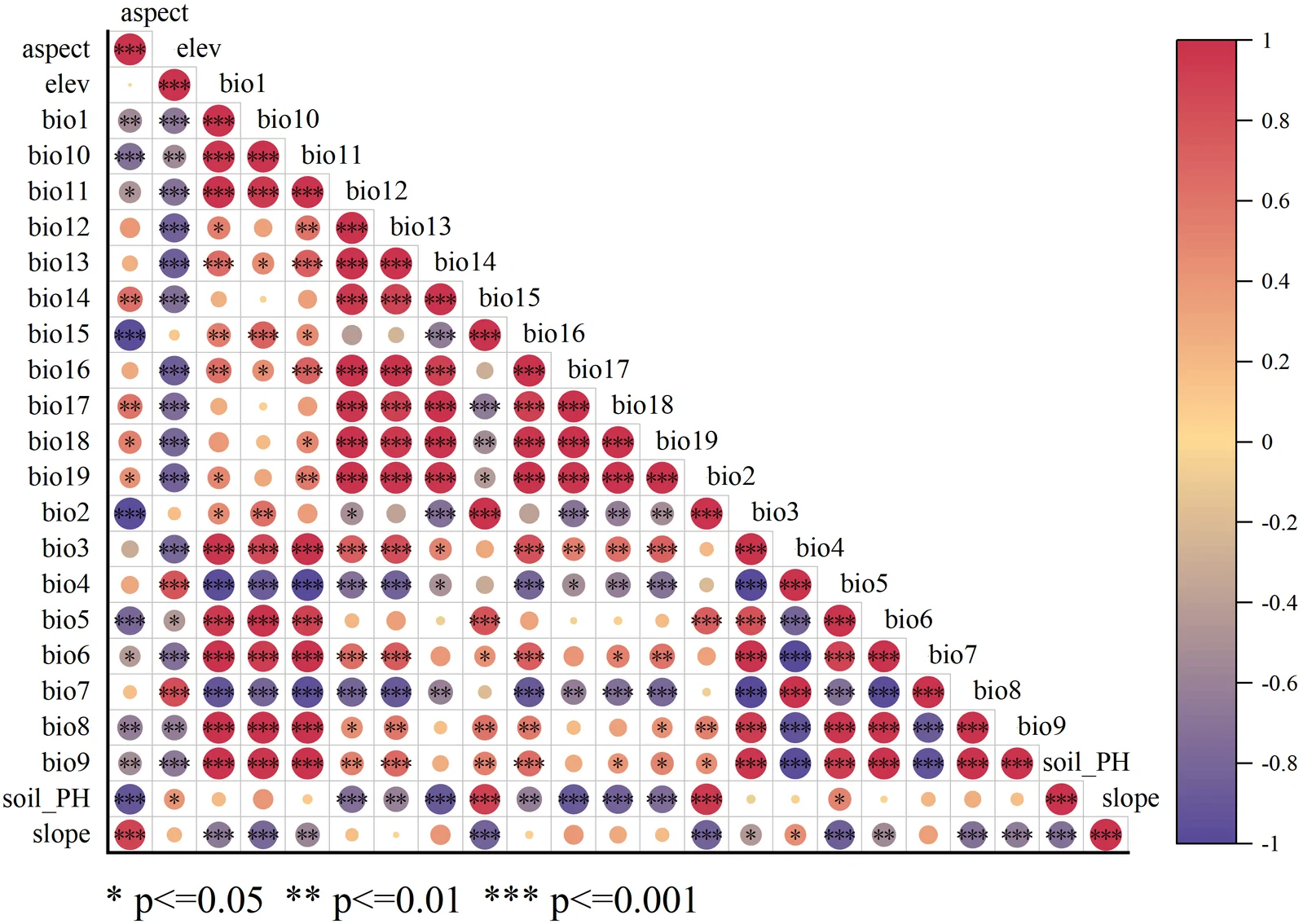
Correlation analysis of 23 environmental variables.

To enhance the accuracy of the model outputs, twenty occurrence records of *Coptis teeta* Wall. within Yunnan Province, along with contemporary bioclimatic data, were employed to perform initial simulations using MaxEnt software. This process determined the percent contribution of each environmental variable to the model. Subsequently, based on these contribution values, a Pearson correlation analysis was conducted for the 23 environmental variables using R software to evaluate and mitigate multicollinearity (**Figure 2**). Variables with high collinearity (|r| ≥ 0.8) were considered redundant. When two highly correlated variables showed similar ecological importance, the variable with higher contribution and greater ecological interpretability was retained for subsequent modeling.

By analyzing the contribution rates of the 23 environmental variables and incorporating the results of the correlation analysis, six principal drivers governing the potential suitable habitat distribution of *Coptis teeta* Wall. were identified: precipitation of the warmest quarter (Bio18), slope, temperature seasonality (Bio4), elevation, temperature annual range (Bio7), and minimum temperature of the coldest month (Bio6) (**Table 3**).Although some differences existed between percent contribution and permutation importance rankings, these two metrics evaluate variable importance from different perspectives. Therefore, both metrics were considered when interpreting the ecological effects of environmental variables.

**Table 3:** Six selected dominant environmental factor parameters.

Environmental factor	Percentage contribution (%)	Replacement importance
Bio18 (Precipitation in the warmest season)	42.5	7.1
Slope	16.7	0.4
Bio4 (Temperature Seasonality)	10.4	0.4
Elevation	9.1	1
Bio7 (Annual Temperature Range)	1.5	53.9
Bio6 (Lowest Temperature in Coldest Month)	2.1	22.9

### 2.4 MaxEnt model prediction

A total of 20 occurrence records for *Coptis teeta* Wall. were integrated into the MaxEnt model along with the six selected dominant environmental variables (Bio18, Slope, Bio4, Elevation, Bio7, and Bio6) to simulate potential suitable habitats. Response curves were generated to evaluate niche preferences across different temporal scales, including the past (LGM and mid-Holocene), contemporary, and future periods under four Shared Socioeconomic Pathways (SSPs). To ensure model robustness, ten replicates were performed using a cross-validation approach, yielding Receiver Operating Characteristic (ROC) curves and predicted distribution maps. Model performance was evaluated using the Area Under the Curve (AUC), where the X-axis represents 1- specificity and the Y-axis represents sensitivity. Generally, an AUC value exceeding 0.9 is considered to indicate excellent predictive performance (Shu et al., 2023).

### 2.5 Technical approach

**Figure 3:**
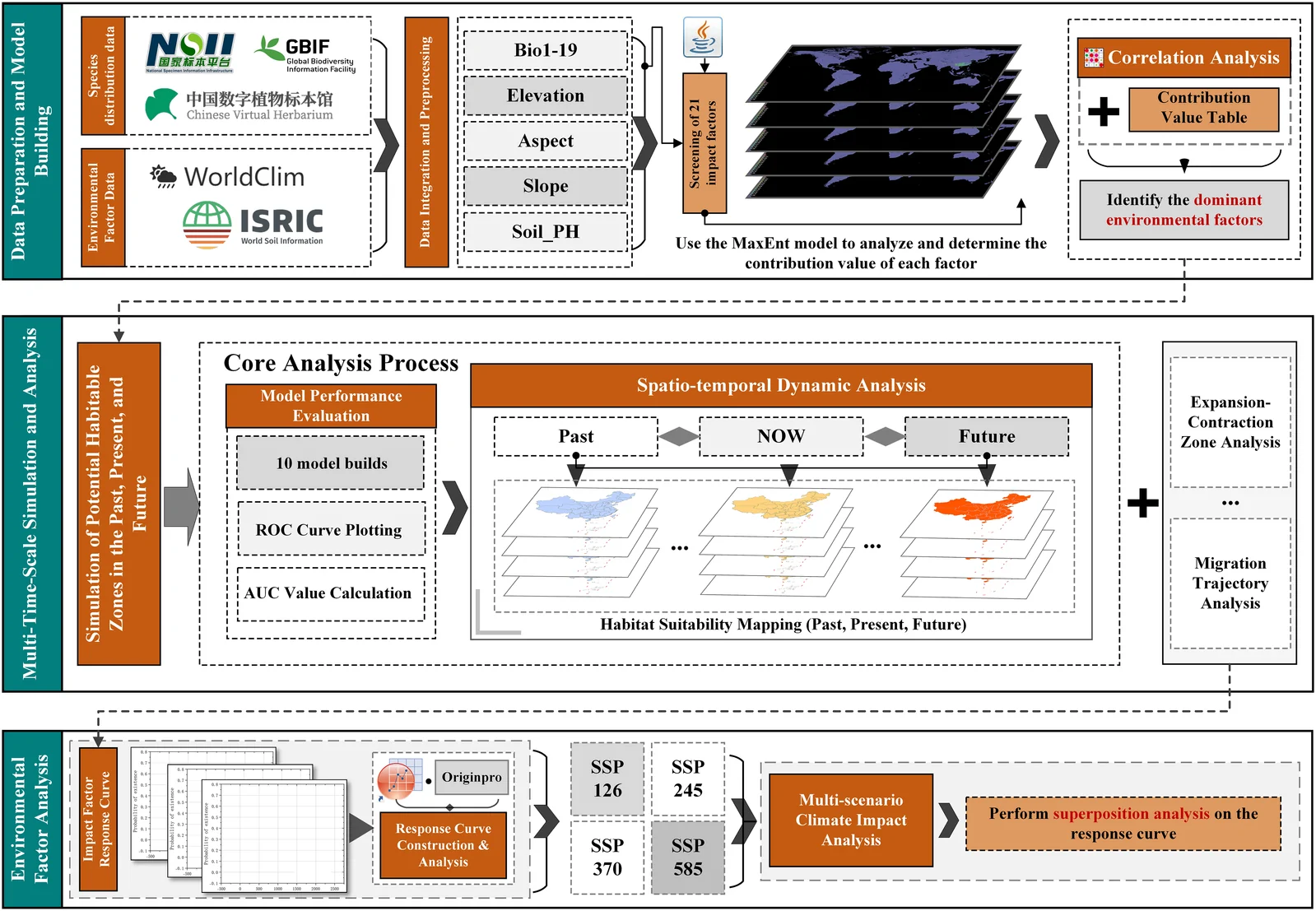
Technology roadmap.

This study presents a systematic technical framework for predicting the potential geographic distribution of *Coptis teeta* Wall. First, occurrence records were compiled from several databases, including NSII, GBIF, and CVH, and subjected to spatial thinning to mitigate sampling bias. Concurrently, bioclimatic variables were acquired from the WorldClim database. To enhance model parsimony and predictive accuracy, dominant variables were selected through Pearson correlation analysis and percent contribution assessments, effectively addressing multicollinearity among environmental variables. Subsequently, the MaxEnt algorithm was employed to simulate potential habitat distribution, with model performance evaluated via 10-fold cross-validation and the Area Under the Curve (AUC) metric. Based on the visualized suitable habitat distributions, spatiotemporal dynamics under various climate scenarios were further quantified through range expansion-contraction analysis and the tracking of centroid migration trajectories. This integrated approach elucidates the ecological requirements of *Coptis teeta* Wall. and assesses the consistency and sensitivity of dominant environmental variables across different environmental gradients (**Figure 3**).

## 3 Results and analysis

### 3.1 MaxEnt model accuracy results

**Figure 4:**
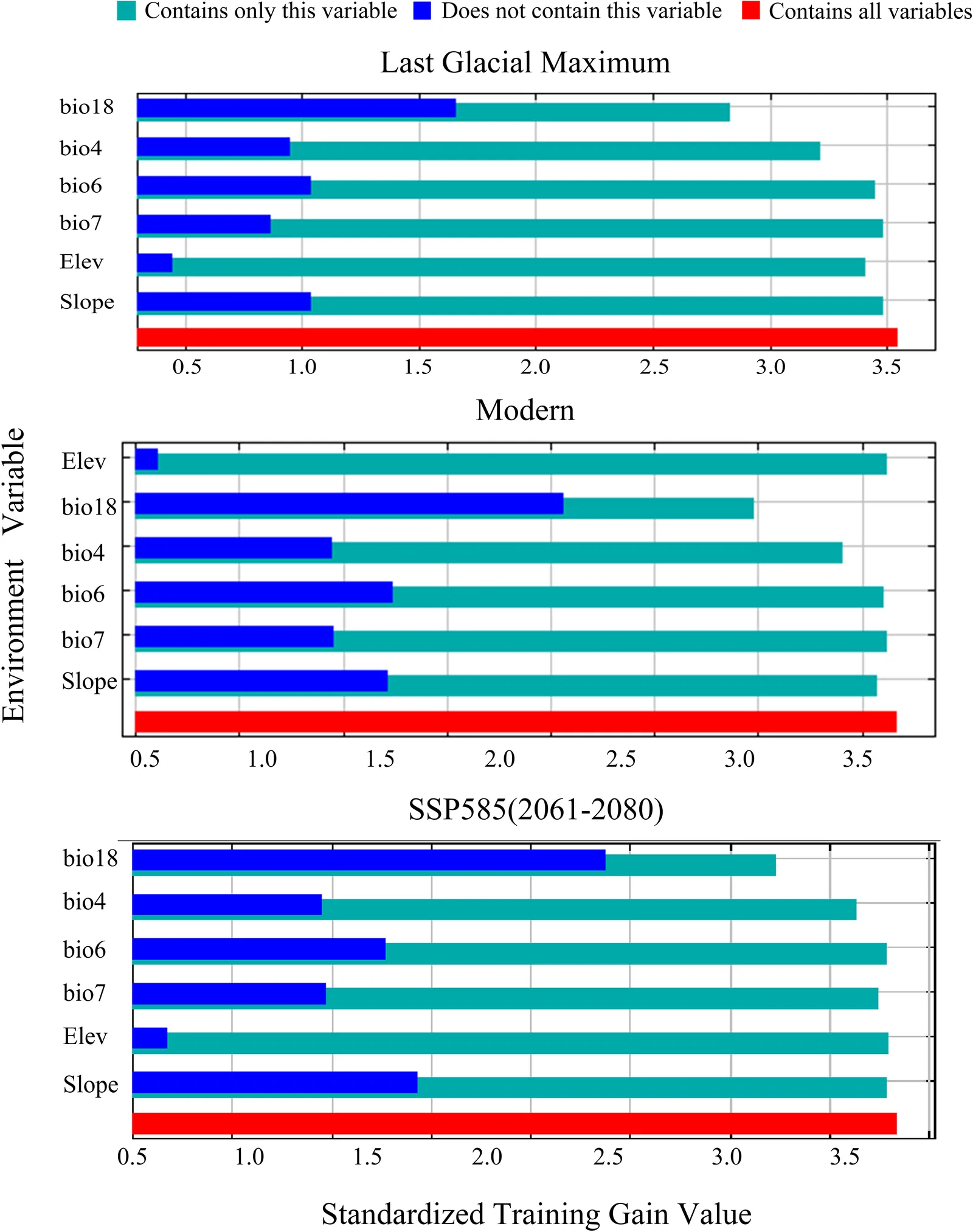
Jackknife test analysis of the six dominant environmental factors across three key periods.

Utilizing the selected environmental variables, the Area Under the Curve (AUC) values derived from the MaxEnt model for the three primary temporal periods consistently exceeded 0.9, demonstrating excellent predictive performance and model robustness (**Figure 4**).

### 3.2 Analysis of potential distribution areas for *Coptis teeta* Wall

#### 3.2.1 Spatiotemporal dynamics analysis of *Coptis teeta* Wall from past to present

**Figure 5:**
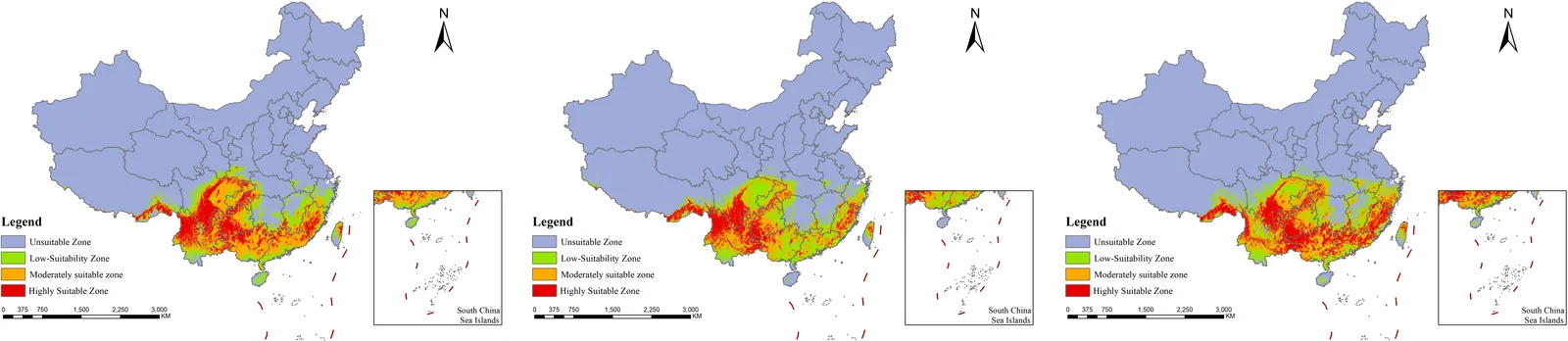
**(a)** Last Glacial Maximum suitable area map **(b)** Mid-Holocene suitable area map **(c)** Modern suitable area map. Distribution of *Coptis teeta* Wall suitable areas from past periods to the present.

Spatial analysis of the habitat suitability distributions for *Coptis teeta* Wall. was performed using ArcGIS. The ASCII output files corresponding to various temporal periods—specifically the past (LGM and mid-Holocene), contemporary, and future scenarios—were imported into the GIS environment. Habitat suitability indices (HSI) were subsequently reclassified into four grades based on established thresholds: unsuitable (0–0.2), low suitability (0.2–0.4), moderately suitable (0.4–0.6), and highly suitable (0.6–1.0). To align with the study’s focus on China, the resulting layers were masked using national administrative boundaries to generate potential distribution maps constrained to the Chinese territory (**Figure 5**).

Analysis reveals that during the Last Glacial Maximum (LGM), the primary suitable habitat for *Coptis teeta* Wall. was principally concentrated in central-southern Sichuan, central-northern Yunnan, central-western Guizhou, eastern Chongqing, northern Guangxi, Guangdong, central-southern Fujian, northeastern Taiwan, and southeastern Tibet. By the mid-Holocene, the suitable habitat remained largely stable across these regions, although significant contractions were observed in Guangxi, Guangdong, and Fujian provinces. In the contemporary period, the suitable habitat is primarily distributed in southeastern Tibet, southern Sichuan, and western Guizhou, while highly suitable areas have re-emerged in Guangxi, Guangdong, and Fujian. Overall, the potential suitable habitat for this species across these three temporal stages exhibited a discernible eastward shift from southwestern China toward the southeastern coastal regions.

To facilitate quantitative analysis of the suitable habitat areas, the reclassified distribution maps were further processed in ArcGIS. The suitability layers were binarized into a Boolean format (0, 1), where 0 denotes unsuitable regions and 1 represents suitable habitats. In this study, the total suitable area was defined as the sum of moderately suitable (0.4–0.6) and highly suitable (0.6–1.0) zones. Utilizing the ArcGIS Spatial Analyst tools, the extent of these binarized suitable zones was computed for three distinct periods: The Last Glacial Maximum (LGM) (143.52×10^4^km^2^), the mid-Holocene (115.92×10^4^km^2^), and the contemporary period (151.05×10^4^km^2^). Overall, from the LGM to the contemporary period, the suitable habitat area for *Coptis teeta* Wall. exhibited a fluctuating trend, characterized by an initial contraction followed by a subsequent expansion. The contemporary suitable area is currently the most extensive, representing increases of 5.25% and 30.31% relative to the LGM and the mid-Holocene, respectively.

Further quantification of the individual suitable habitat zones across different temporal scales elucidated their specific evolutionary trends. Analysis conducted in ArcGIS demonstrated that the highly suitable zone attained its maximum extent in the contemporary period, reaching 65.71×10^4^km^2^, which represents expansions of 12.37% and 40.75% relative to the Last Glacial Maximum (LGM) and mid-Holocene, respectively. However, the highly suitable area for *Coptis teeta* Wall. contracted during the mid-Holocene by 20.16% compared to the LGM (**Table 4**).

**Table 4:** Changes in the area of suitable habitat for *Coptis teeta* Wall in different periods.

Period	Area changes of each suitable habitat zone across periods (×10^4^ km^2^)		
	High-suitability zone	Moderate habitat zone	Low-suitable zone
Last Glacial Maximum	58.4704	85.0460	58.4704
Mid-Holocene	46.6830	69.2375	74.4509
Modern	65.7058	85.3484	68.9540

#### 3.2.2 Prediction of future suitable areas under multiple scenarios using

**Figure 6:**
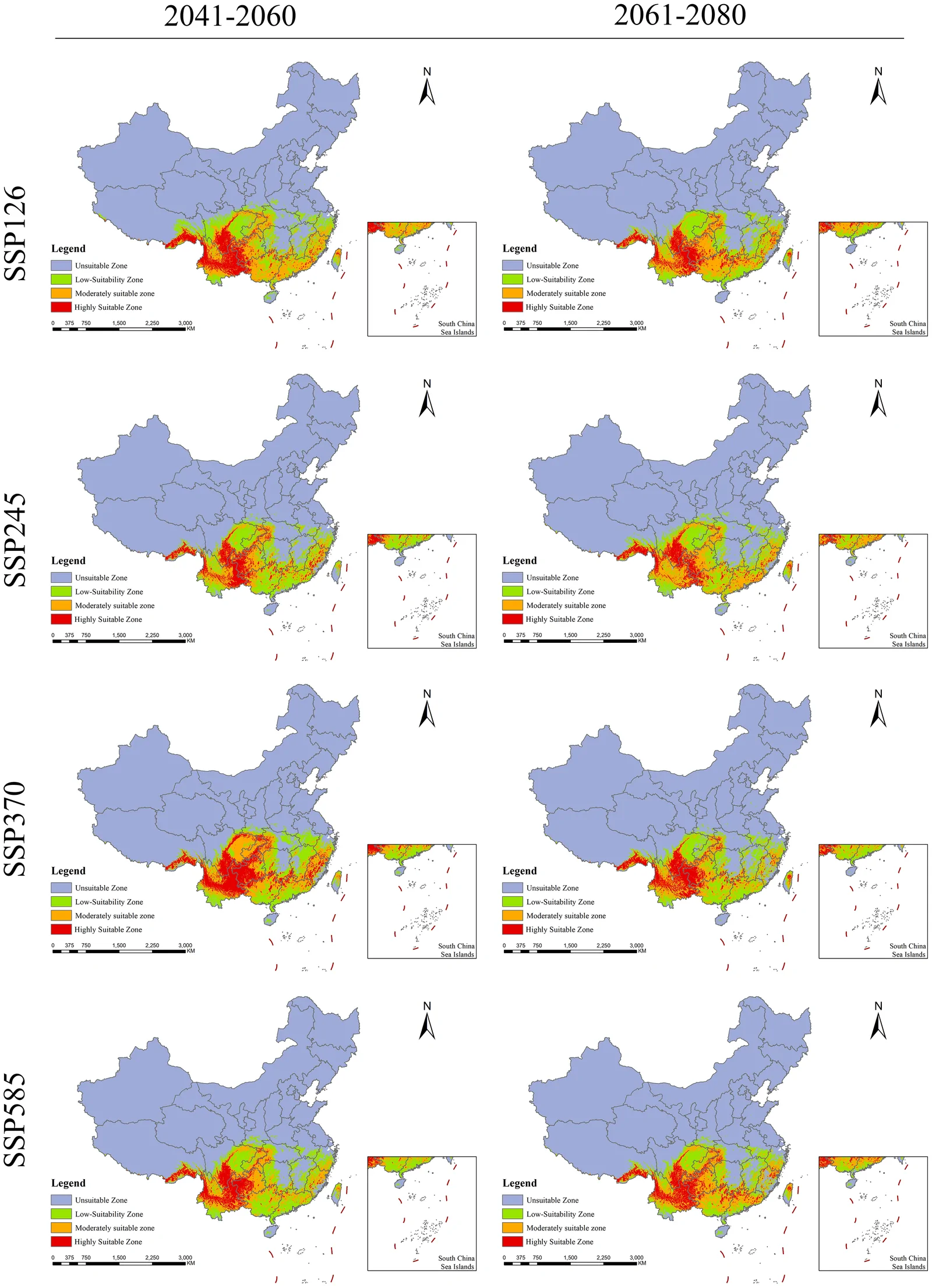
Predicted distribution maps of suitable areas for *Coptis teeta* Wall at different stages under future climate scenarios.

**Figure 7:**
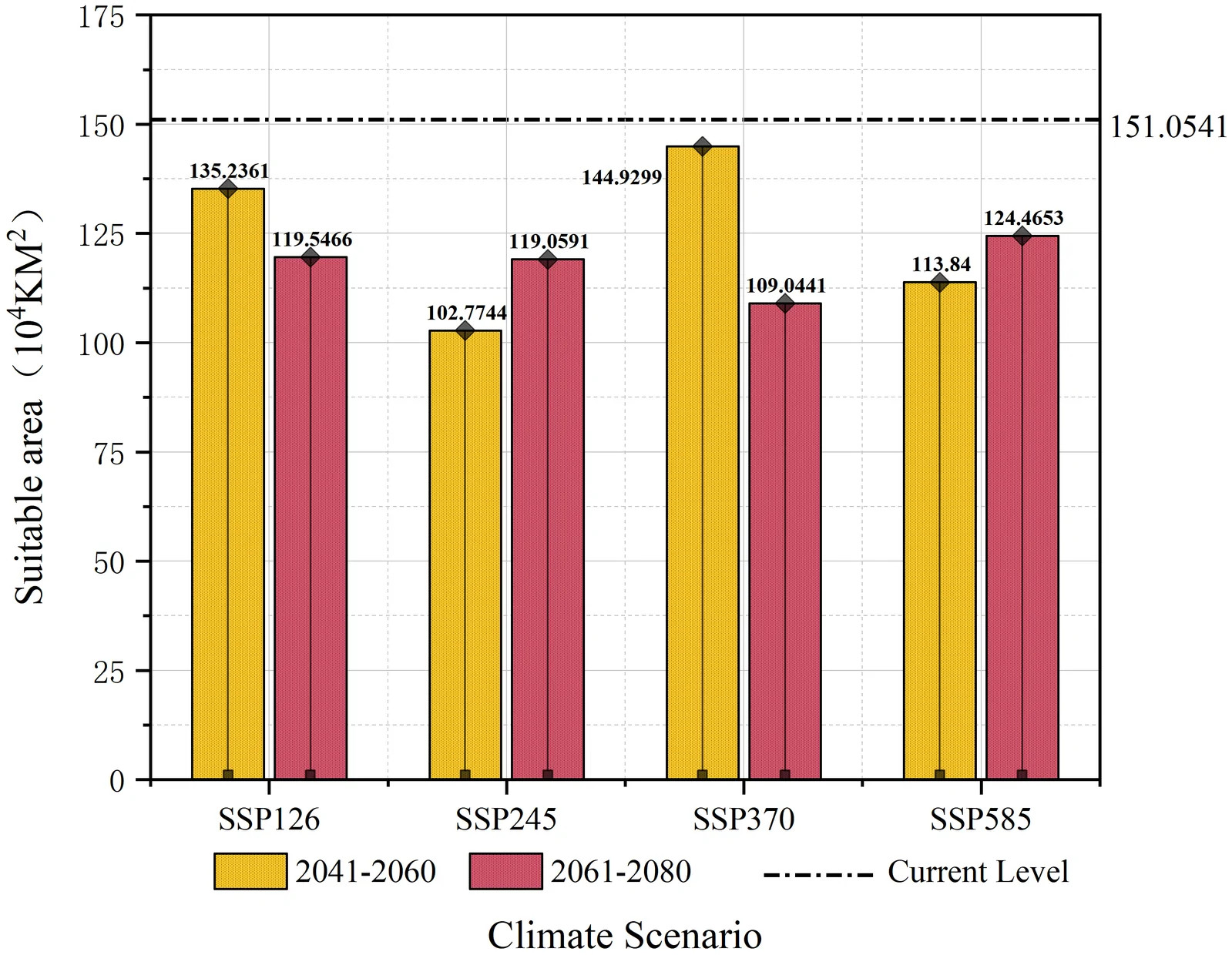
Statistical analysis of the suitable habitat area for *Coptis teeta* Wall. under current and future climate scenarios (SSPs).

ArcGIS 10.8 under four Shared Socioeconomic Pathways (SSP126, SSP245, SSP370, and SSP585) across two future periods: the 2050s (2041–2060) and 2070s (2061–2080). Compared to contemporary conditions, the suitable area for this species exhibits a general contraction under all selected climate scenarios and time periods. Under the low-emission SSP126 scenario, the suitable area consistently declines to 135.24×10^4^km^2^ and 119.55×10^4^km^2^ in the 2050s and 2070s, corresponding to reduction rates of 10.47% and 20.86%, respectively. Under the SSP245 and SSP585 scenarios, the habitat extent exhibited non-monotonic fluctuations, characterized by an initial reduction followed by expansion. Specifically, under SSP245, the suitable areas were 102.77×10^4^km^2^ (−31.96%) and 119.06×10^4^km^2^ (−21.16%). Under SSP585, the areas totaled 113.84×10^4^km^2^ and 124.47×10^4^km^2^, representing contractions of 24.63% and 17.60% relative to contemporary areas. Finally, under the SSP370 scenario, a pronounced declining trend was observed, with suitable areas decreasing to 144.93×10^4^km^2^ (−4.05%) and 109.04×10^4^km^2^ (−27.55%) across the two periods (**Figures 6**, **7**).

### 3.3 Analysis of expansion and contraction zones for future suitable habitats of *Coptis teeta* Wall

**Figure 8:**
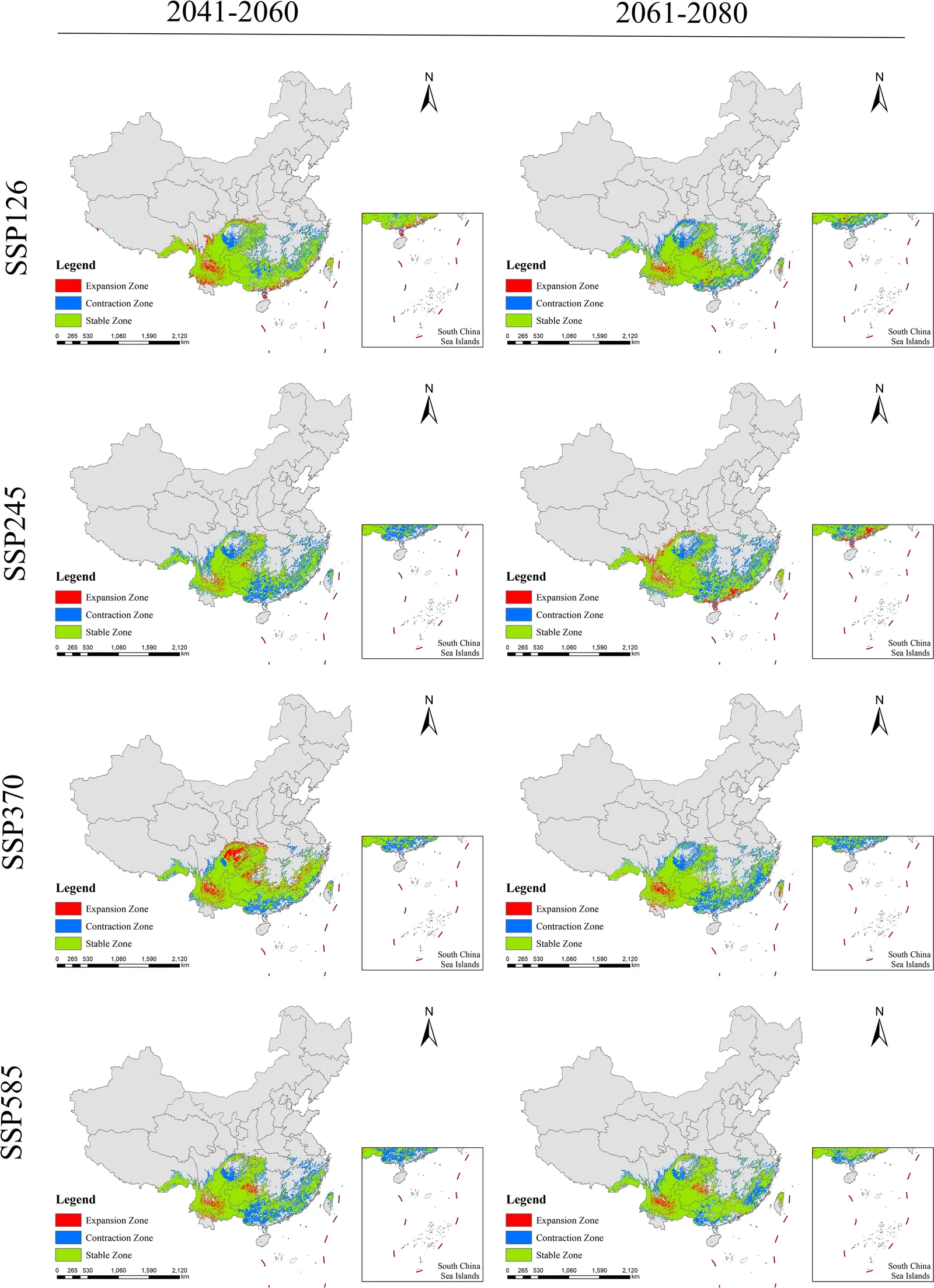
Expansion and contraction of suitable areas for *Coptis teeta* Wall at different stages under future climate scenarios.

By synthesizing the past (LGM, mid-Holocene), contemporary, and future climate scenarios, a comprehensive map of habitat range dynamics—including contraction, expansion, and stable zones—was generated for *Coptis teeta* Wall. (**Figure 8**). The potential climatic refugia (stable habitats) are projected to persist primarily in central, southeastern, and northeastern Sichuan; central and western Guizhou; and most of Yunnan (excluding the southern region), with additional stable distributions in Guangxi, Guangdong, and central Fujian. In southeastern Sichuan, habitat expansion is projected exclusively under the SSP370 scenario during the 2050s (2041–2060), whereas contraction remains prevalent across all other scenarios and temporal periods. Remaining contraction zones are primarily clustered in southeastern coastal areas, including Guangxi, Guangdong, and Fujian, while expansion zones are relatively fragmented and principally located on the periphery of stable areas.

Quantitative analysis using ArcGIS 10.8 revealed that under the low-emission SSP126 scenario, *Coptis teeta* Wall. undergoes sustained habitat contraction. From the 2050s to the 2070s, the stable habitat area is projected to decrease by approximately 8.9×10^4^km^2^, while the contracted area expands by 28.8%. Under the medium-emission SSP245 scenario, the stable zone reaches its minimum extent among the four scenarios during the 2050s (97.58×10^4^km^2^) before rebounding to 106.17×10^4^km^2^ in the 2070s. Under the medium-to-high emission SSP370 scenario, the 2050s period exhibits the largest stable area (124.20×10^4^km^2^); however, the contraction zone is projected to increase substantially by 22.07×10^4^km^2^ by the 2070s, while the expansion zone diminishes to 6.58×10^4^km^2^. Finally, under the high-emission SSP585 scenario, the stable zone expands by 10.71 ×10^4^km^2^ between the two periods, while the contraction zone decreases by 10.70×10^4^km^2^, with the expansion zone remaining constant at approximately 9×10^4^km^2^ (**Table 5**).

**Table 5:** Area statistics of spatial range dynamics (expansion, contraction, and stability) for *Coptis teeta* Wall under different future climate scenarios and time periods.

Four climate scenarios	Year	Distribution range dynamics	Area/10^4^km^2^
SSP126	2041–2060	Stable Zone	119.6902
		Contraction Zone	30.9444
		Expansion Zone	15.0432
	2061-2080	Stable Zone	110.7807
		Contraction Zone	39.8653
		Expansion Zone	8.4806
SSP245	2041-2060	Stable Zone	97.5761
		Contraction Zone	53.0453
		Expansion Zone	4.9225
	2061-2080	Stable Zone	106.1663
		Contraction Zone	44.3928
		Expansion Zone	12.4186
SSP370	2041-2060	Stable Zone	124.2008
		Contraction Zone	26.4282
		Expansion Zone	20.3833
	2061-2080	Stable Zone	102.3455
		Contraction Zone	48.4989
		Expansion Zone	6.5834
SSP585	2041-2060	Stable Zone	103.9667
		Contraction Zone	46.6584
		Expansion Zone	9.5879
	2061-2080	Stable Zone	114.6752
		Contraction Zone	35.9632
		Expansion Zone	9.4084

The spatiotemporal dynamics of the suitable habitat for *Coptis teeta* Wall. under various Shared Socioeconomic Pathways (SSPs) exhibit marked heterogeneity and non-monotonic evolutionary trajectories. Under the low-emission scenario (SSP126), the species encounters persistent habitat degradation pressure, characterized by a progressive annual shrinkage of stable refugia, coupled with a continuous expansion of contraction zones and a significant attenuation of expansion potential. In stark contrast, the medium-emission (SSP245) and high-emission (SSP585) scenarios demonstrate notable habitat resilience and restoration trends, where the extent of stable zones increases from the mid- to late-term, and habitat contraction is effectively mitigated. However, under the medium-to-high emission scenario (SSP370), the suitable habitat undergoes a bifurcated “early stability followed by late-stage collapse” pattern: while maintaining stable distributions in the initial phase, it experiences a severe habitat crisis in the later stage, marked by the simultaneous reduction of stable and expansion areas and a sharp spike in habitat loss. Overall, *Coptis teeta* Wall. exhibits profound sensitivity to climate pathways, with its long-term habitat stability displaying divergent and multifaceted response patterns under varying emission intensities.

### 3.4 Center of gravity migration analysis

**Figure 9:**
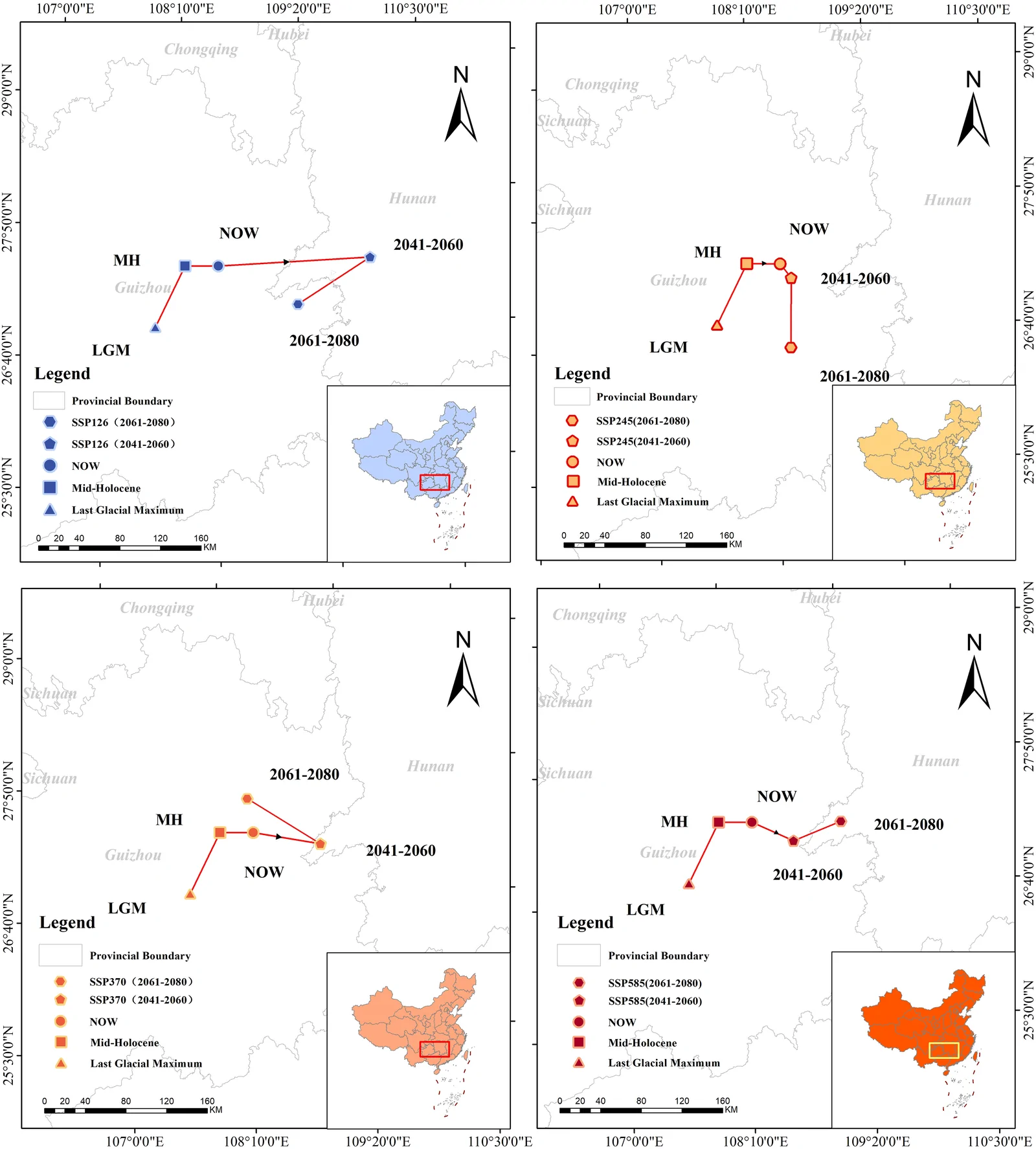
Spatiotemporal migration trajectories of the *Coptis teeta* Wall centroid from the Last Glacial Maximum to the late 21st century under four SSP scenarios.

Based on the habitat suitability data for *Coptis teeta* Wall. during the Last Glacial Maximum (LGM), mid-Holocene, contemporary, and projected future periods, ArcGIS 10.8 was utilized to analyze the migration trajectories of the species’ distribution centroid. Results indicate that from the LGM to the 2080s, the centroid underwent complex spatial shifts(**Figure 9**). During the historical evolution phase, the centroid exhibited an overall northeastward shift: originating in central Guizhou (107.68° E, 26.83° N) during the LGM, migrating to central-eastern Guizhou (108.02° E, 27.35° N) by the mid-Holocene, and reaching northeastern Guizhou (108.34° E, 27.33° N) in the contemporary period.

For the future period (2041–2080), the centroid migration patterns diverge significantly under different climate scenarios. Under SSP126, the centroid initially displaces eastward to western Hunan (109.84° E, 27.30° N) in the 2050s, before retrogressing to Guizhou (109.10° E, 26.94° N) in the 2070s, with a cumulative migration distance of 334.34 km. Under SSP245, the centroid shifts southeastward to central-eastern Guizhou (108.44° E, 27.20° N) in the 2050s and subsequently veers southward (108.39° E, 26.59° N) by the 2070s, spanning 185.89 km. Under SSP370, the centroid moves southeastward into Hunan (108.99° E, 27.19° N) by the 2050s, followed by a substantial northwesterly recoil by the 2070s, returning to Guizhou (108.31° E, 27.63° N) with a distance of 251.24 km. Under the SSP585 ultra-high emissions scenario, the centroid exhibits a stable, sustained eastward drift, reaching 108.74° E, 27.14° N in the 2050s and 109.21° E, 27.28° N in the 2070s, totaling 194.90 km. Overall, the distribution centroid of *Coptis teeta* Wall. is projected to undergo varying degrees of oscillatory or unidirectional displacement, primarily along the border regions of northeastern Guizhou and western Hunan.

Historically, the distribution centroid of *Coptis teeta* Wall. migrated from central Guizhou through the east-central region toward the northeast, ultimately stabilizing in northeastern Guizhou in contemporary times. Entering the future period (2050s–2070s), the centroid displacement exhibits pronounced divergence across different Shared Socioeconomic Pathways (SSPs). Under the SSP126 and SSP370 scenarios, the centroid displays significant oscillatory fluctuations, initially shifting eastward or southeastward into Hunan Province prior to a distinct retrogression or a substantial northwesterly shift in the long term. This spatial instability underscores the species’ sensitivity and vulnerability to uncertain climate fluctuations. In contrast, under the medium-emission scenario (SSP245) and the very high-emission scenario (SSP585), the centroid exhibited a southward deflection and a persistent eastward trajectory, respectively.

### 3.5 Analysis of environmental variables affecting

**Figure 10:**
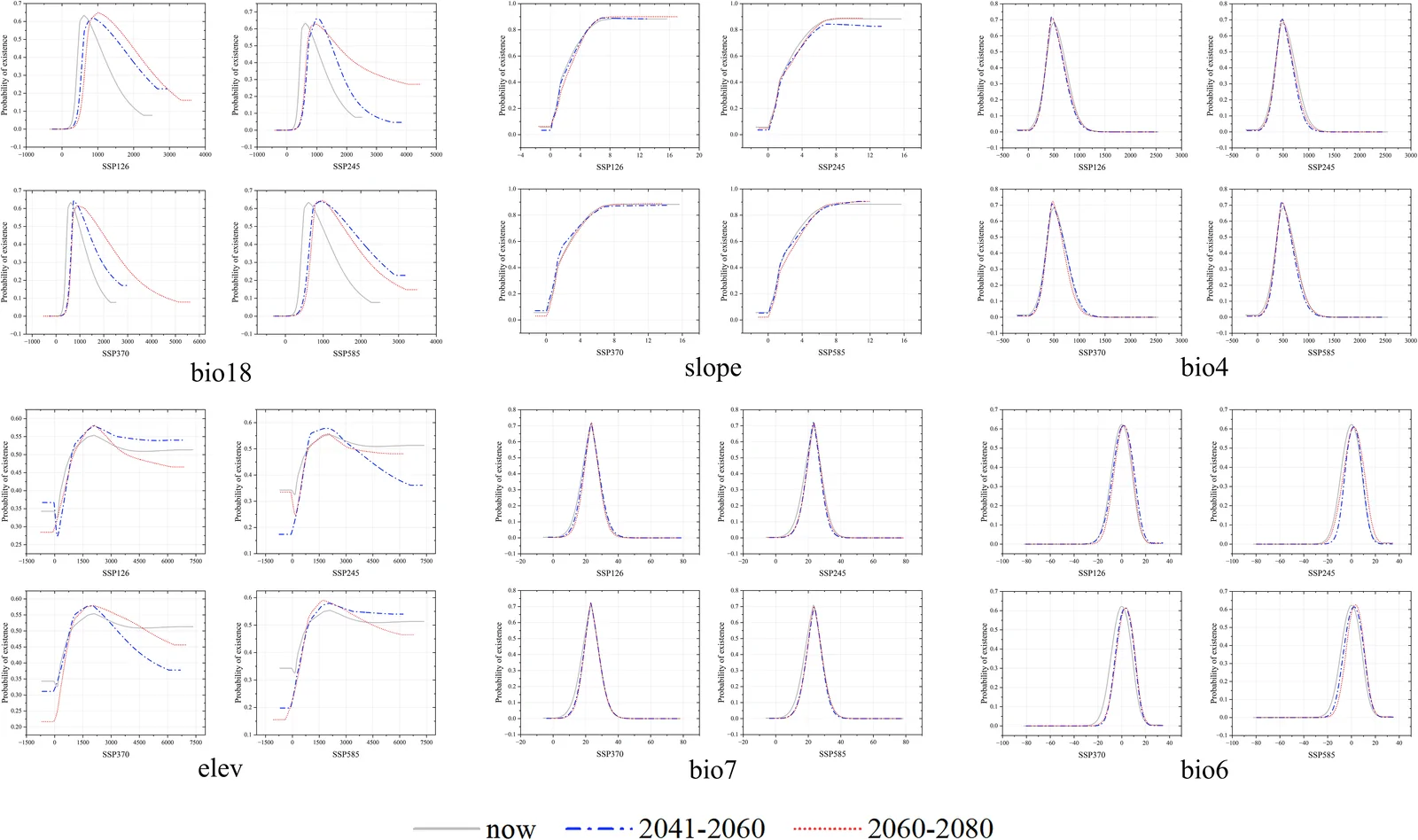
Spatiotemporal migration trajectories of the *Coptis teeta* Wall centroid from the Last Glacial Maximum to the late 21st century under four SSP scenarios.

The response curves for the six dominant bioclimatic and topographic variables affecting *Coptis teeta* Wall. were visualized using OriginPro 2024 (**Figure 10**). Regarding Bio18 (precipitation of the warmest quarter), the contemporary adaptation niche is relatively narrow and concentrated in low-precipitation regions. However, across all future SSP scenarios, the response curves exhibit a pronounced rightward shift and a broadening of the suitability range. This shift indicates that future climate change will significantly increase precipitation during the warmest season within the species’ range, forcing *Coptis teeta* Wall. to adapt to higher precipitation intensities. Under contemporary conditions, the optimal Bio18 value is 619 mm, corresponding to a 63% suitability probability; notably, the species displays higher tolerance under the SSP126, SSP245, and SSP370 scenarios by the 2070s (2061–2080).

The slope response curve remains consistent across all scenarios, confirming that topographic features constitute permanent environmental constraints. The contemporary optimal slope is identified at 8°, corresponding to a peak suitability of 90%. For Bio4 (temperature seasonality), the response curves follow a unimodal distribution across all scenarios, with contemporary optimal suitability peaking between 450 and 500 (~70% probability). In the late-term (2070s) under the SSP370 scenario, the optimal value shifts slightly leftward, suggesting that extreme thermal instability may contract the plant’s climatic niche. The elevation (Elev) response curve typically exhibits a rapid initial ascent to a peak followed by gradual stabilization. The contemporary optimal zone spans 1500 to 2500 m, with a suitability probability of approximately 55%. In the near-term (2041–2060), future scenarios project peak elevations approximately 1750 m higher than the contemporary peak. However, by the 2070s under SSP126 and SSP585, the peak suitability falls below these near-term levels. Crucially, the suitability probability beyond 4500 m in the long term is lower than at present, suggesting a “summit squeeze” effect where upslope migration is restricted by both physiological thresholds and the diminishing land area available at high altitudes.

Bio7 (temperature annual range) and Bio6 (minimum temperature of the coldest month) also exhibit unimodal distributions. The contemporary optimal Bio7 range is 22 °C–26 °C (optimum ~23 °C), with a 70% suitability probability. The high overlap between historical and future Bio7 curves indicates that annual temperature fluctuations will remain a critical limiting factor. For Bio6, the contemporary optimal value is 0.78 °C (range 0 °C–2 °C). In all future scenarios, the Bio6 peak shifts rightward, suggesting that the modeled suitable habitats may become associated with relatively warmer winter conditions in the future. Regions experiencing substantial winter warming may potentially alter the thermal conditions associated with the species’ growth and regeneration processes; however, this hypothesis requires further physiological validation, potentially leading to localized range contractions.

Analysis of the response curves for the six dominant factors suggests that climate change will synergistically influence the potential distribution of *Coptis teeta* Wall. through alterations in thermo-hygric regimes and topographic constraints. Regarding Bio18 (precipitation of the warmest quarter), the contemporary suitability breadth is relatively narrow and biased toward lower precipitation levels. However, under future SSP scenarios, the response curve exhibits a significant rightward shift and pronounced broadening, particularly in the long-term (2070s). This suggests that the modeled climatic suitability of Coptis teeta Wall. may expand under future precipitation conditions. The slope factor displays highly consistent response characteristics across all scenarios and temporal scales, with the optimal gradient remaining stable at approximately 8°. This underscores that topographic features function as fundamental and immutable constraints on the distribution of *Coptis teeta* Wall. Furthermore, the response curves for Bio4 (temperature seasonality) and Bio7 (temperature annual range) both display unimodal Gaussian-like distributions, with optimal ranges concentrated at 450–500 and 22°–26 °C, respectively. Their stable morphology indicates that temperature fluctuation amplitudes impose strong stabilizing constraints on the species’ ecological niche. The elevation (Elev) response curve highlights marked altitudinal zonation, with the contemporary optimal habitat identified between 1500–2500 m. Future near-term scenarios (2050s) project an upslope migration of the peak suitability, while long-term projections indicate a significant decline in habitat probability at high elevations. Finally, the Bio6 (minimum temperature of the coldest month) response curve shifts rightward in its entirety, suggesting that future climatic suitability may gradually shift toward areas with higher minimum temperatures during the coldest month. The contraction of its low-temperature tolerance threshold may result in localized range shifts and potential habitat fragmentation.

## 4 Discussion

As a globally Critically Endangered (CR) species and a Class II state-protected plant in China, *Coptis teeta* Wall. is endemic to southwestern China. It possesses a notably narrow ecological niche, primarily thriving on the steep slopes of moist evergreen broadleaf forests and mixed coniferous-broadleaf forests (Huang et al., 2004; Wang et al., 2013). Its intrinsic reproductive characteristics intensify the spatial and genetic isolation between extant populations in Yunnan. Specifically, investigations into its reproductive biology have revealed extremely inefficient seed dispersal and exceptionally low seedling recruitment rates (Yang et al., 2012). Methodologically, this study employed the MaxEnt (Maximum Entropy) model, a widely recognized tool for predicting the suitable habitats of endangered medicinal plants. The model integrates environmental variables with species occurrence records, and its robustness has been validated by prior research on congeneric species (Liu et al., 2016). While previous studies on *Coptis teeta* Wall. in Yunnan have focused on cultivation techniques (Li, 2013; Zhang, 2018), community dynamics, and pest control (Tang, 2013; Wang et al., 2013), comprehensive research regarding its potential distribution patterns and dominant environmental drivers remains limited. This study provides theoretical support for the conservation and sustainable management of future suitable habitats for *Coptis teeta* Wall. through predictive analysis of its geographic distribution.

### 4.1 Climatic and topographic drivers of *Coptis teeta* Wall distributions

Our findings identify precipitation of the warmest quarter (Bio18) as the primary driver for the distribution of *Coptis teeta* Wall. (contribution rate: 42.5%), closely aligning with its hygrophilous ecological traits. Under future SSP scenarios, the rightward shift in response curves signifies that the species must adapt to intensified precipitation. Additionally, slope (~8°) and elevation (1500-2500m) exhibit high topographic stability, limiting lateral migration and rendering the species highly susceptible to the “summit squeeze” effect under progressive warming (Hoover et al., 2010; Peters and Darling, 1985).

### 4.2 Historical and future changes in habitat suitability

Spatiotemporal evolution reveals that suitable habitat areas decreased since the Last Glacial Maximum (LGM) before expanding to a contemporary peak (151.05×10^4^km^2^); however, widespread contraction is projected for the future. It should be noted that the predicted modern suitable areas represent potential climatic and topographic suitability, rather than the actual distribution range of *Coptis teeta* Wall. The predicted range is relatively broad; since the model primarily relies on climatic and topographic conditions to predict environmentally similar areas, the establishment of actual populations is further constrained by factors such as habitat specificity, microenvironmental conditions, dispersal limitations, low seedling regeneration rates, and biotic interactions. Therefore, areas identified as suitable outside the currently recorded distribution range represent areas with potential environmental compatibility, rather than existing habitats of *Coptis teeta* Wall.

### 4.3 Future southeastward shift and potential climate refugia

Contrary to the traditional paradigm of “migration toward higher altitudes and latitudes (Zhao et al., 2021),” this study reveals a discernible tendency toward southeastward coastal migration. The distribution centroid is projected to fluctuate primarily along the border region of Guizhou and Hunan, reflecting the species’ pursuit of microclimatic refugia that offer optimal thermo-hygric coupling. However, the predicted shift of the center of gravity toward the Guizhou–Hunan border does not imply that conservation priorities will immediately shift. This shift in focus merely reflects the spatial distribution balance of predicted suitable habitats under different climate scenarios; conservation planning must also consider the survival of existing populations, genetic diversity, habitat integrity, and the feasibility of management measures. In addition, anthropogenic pressures such as overharvesting for medicinal use and habitat fragmentation should also be considered, as these factors may substantially influence population persistence but were not incorporated into the current climatic niche model. Therefore, based on existing population records in the Sichuan-Guizhou-Yunnan region, this area remains critically important for *in situ* conservation, while the Guizhou–Hunan border region may represent a potential future climate refuge and requires further monitoring.

This south-easterly shift is closely linked to the species strong dependence on water availability during the warmest season. As a moisture-loving understory herb inhabiting humid evergreen broad-leaved forests and mixed forests, *Coptis teeta* Wall. requires relatively stable moisture conditions to support vegetative growth, seed germination, and seedling establishment. Reduced precipitation during the warm season will exacerbate soil moisture stress and limit population regeneration, whereas, according to future climate scenarios, northeastern Guizhou and western Hunan may maintain more favorable water and heat conditions. Therefore, the predicted migration pattern likely reflects the redistribution of suitable water and heat environments rather than a simple response to warming.

### 4.4 Ecological risks under future climate change

Under the SSP370 scenario (2061-2080), habitats face severe collapse, with temperature seasonality (Bio4) and annual range (Bio7) causing significant niche contraction. Furthermore, winter warming (Bio6) may jeopardize natural reproduction by disrupting vernalization and dormancy requirements. When combined with low seed dispersal efficiency and sparse seedling recruitment, habitat fragmentation and contraction will substantially heighten extinction risks. Previous research suggests that global warming induces desiccation in low-elevation habitats, while shifts in precipitation patterns in alpine zones disrupt existing thermo-hygric equilibria (Song et al., 2021).

### 4.5 Uncertainty and limitations

In summary, this study employed the MaxEnt model and WorldClim datasets to reconstruct the potential habitat distribution patterns of *Coptis teeta* Wall. across past, present, and future periods, thereby providing a theoretical foundation for the conservation of this critically endangered species. Nevertheless, certain uncertainties should be considered when interpreting the predicted habitat distributions. Although the MaxEnt model has been widely applied to species distribution modeling with limited occurrence data, the relatively small number of occurrence records used in this study (20 records) may increase uncertainty in ecological niche estimation and future habitat projections. Furthermore, model evaluation based solely on the AUC may overestimate predictive performance when occurrence data are limited. It is recommended that future studies incorporate larger occurrence datasets and supplementary evaluation metrics (e.g., TSS) to further validate model performance. To minimize potential biases, the occurrence records in this study were carefully screened and spatially filtered, and model reliability was assessed using AUC values, response curves, and jackknife tests. Future research should integrate more extensive field surveys and higher-resolution environmental data, while also addressing the uncertainties associated with the use of a single global climate model (BCC-CSM2-MR). Moreover, studies that integrate multiple CMIP6 models and employ ensemble methods will enhance the reliability and accuracy of predictions.

## 5 Conclusion

Through the construction of MaxEnt models, this study systematically elucidates the mechanisms governing the impact of climate change on the potential habitats of the endangered medicinal herb *Coptis teeta* Wall. Our findings demonstrate that precipitation of the warmest quarter, interacting with specific topographic constraints (slope and elevation), collectively delineate the species’ ecological niche boundaries. Although contemporary suitable areas remain at a historical peak, future warming is projected to trigger severe habitat contraction and a significant centroid shift toward the southeast. Given the species’ limited natural dispersal capacity and the “mountain-top squeeze” effect, which poses a barrier to altitudinal migration, conservation efforts should focus on the existing core distribution areas in the Sichuan–Guizhou–Yunnan region, as well as future potential climate refuges identified along the Guizhou–Hunan border. Furthermore, the strategic deployment of proactive *ex situ* conservation and assisted migration trials is recommended along predicted migration pathways to mitigate extinction risks for this endangered species amid accelerating global warming.

## Data Availability

The raw data supporting the conclusions of this article will be made available by the authors, without undue reservation.

## References

[B1] Aitken S. N. Yeaman S. Holliday J. A. Wang T. Curtis‐McLane S. (2008). Adaptation, migration or extirpation: climate change outcomes for tree populations. Evol. Appl. 1, 95–111. doi: 10.1111/j.1752-4571.2007.00013.x 25567494 PMC3352395

[B2] Bo S. Yu H. Han C. (2024). Adaptability analysis of walnut anthracnose based on MaxEnt model. For. Res. 37, 68–78.

[B3] Breiman L. (2001). Random forests. Mach. Learn. 45, 5–32. doi: 10.1023/a:1010933404324 41886696

[B4] Chi Y. Liu C. Liu W. Tian X. Hu J. Wang B. . (2024). Population genetic variation and geographic distribution of suitable areas of Coptis species in China. Front. Plant Sci. 15, 1341996. doi: 10.3389/fpls.2024.1341996 38567137 PMC10985201

[B5] Dai M. Li X. Wang M. Wen Y. (2022). Potentially suitable area of Cryptomeria japonica var. sinensis and the influence of climate changes on its distribution. J. Northwest. For. Univ. 37, 26–33, 128.

[B6] González-Camacho J. M. Ornella L. Pérez-Rodríguez P. Gianola D. Dreisigacker S. Crossa J. (2018). Applications of machine learning methods to genomic selection in breeding wheat for rust resistance. Plant Genome 11, 170104. doi: 10.3835/plantgenome2017.11.0104 30025028 PMC12962436

[B7] Hamann E. Denney D. Day S. Lombardi E. Jameel M. I. MacTavish R. . (2021). Plant eco-evolutionary responses to climate change: emerging directions. Plant Sci. 304, 110737. doi: 10.1016/j.plantsci.2020.110737 33568289

[B8] Hoover K. J. Poulson S. Biondi F. Underwood J. (2010). “ Eco-hydrological pathways inferred from stable isotopes in a Pinus ponderosa and Pinus monophylla woodland of the Sheep Range, southern Great Basin, USA”, in: 2010 Annual Nevada NSF EPSCoR Climate Change Conference (Las Vegas, NV, United States).

[B9] Huang J. Pei S. Wang Y. (2005). Natural resources and conservation of Coptis teeta. Chin. Tradit. Herb. Drugs 36, 112–115.

[B10] Huang J. Pei S. Zhang M. Mao J. Yang X. (2004). Studies on biological characteristics, ecological habit and geographic distribution of Coptis teeta. Acta Botanica Yunnanica 26, 255–266.

[B11] Kougioumoutzis K. Constantinou I. Panitsa M. (2024). Rising temperatures, falling leaves: predicting the fate of Cyprus’s endemic oak under climate and land use change. Plants 13, 1109. doi: 10.3390/plants13081109 38674518 PMC11053427

[B12] Lenoir J. Gégout J.-C. Marquet P. A. de Ruffray P. Brisse H. (2008). A significant upward shift in plant species optimum elevation during the 20th century. Science 320, 1768–1771. doi: 10.1126/science.1156831 18583610

[B13] Li C. (2013). Key cultivation techniques for Coptis. Sichuan Agric. Sci. Technol. (8), 31–32.

[B14] Liu S. Zhang Z. Chen X. (2025). Prediction of the potential suitable areas for Coptis chinensis in Hubei Province based on the optimized MaxEnt model. J. Yangtze Univ. (Nat. Sci. Edition) 22, 123–130. doi: 10.3969/j.issn.1673-1409.2025.05.015

[B15] Liu X. Yang Y. Song H. Zhang X. Huang L. Wu H. (2016). Cultural regionalization for Coptis chinensis based on 3S technologyplatform I. Study on growth suitability for Coptis chinensis based on ecological factors analysis by Maxent and ArcGlS model. China J. Chin. Materia Med. 41, 3186–3193. doi: 10.4268/cjcmm20161712 28920369

[B16] Liu Y. Wang B. Shu S. Li Z. Song C. Liu D. . (2021). Analysis of the Coptis chinensis genome reveals the diversification of protoberberine-type alkaloids. Nat. Commun. 12, 3276. doi: 10.1038/s41467-021-23611-0 34078898 PMC8172641

[B17] Liu Y. Tan Y.-L. Li Y.-M. Ping Y.-M. He D.-M. Zhang G.-L. . (2024). Conservation and threatened status of plant species with extremely small populations in the karst region of southeastern Yunnan, China. Front. Plant Sci. 15, 1520363. doi: 10.3389/fpls.2024.1520363 39777088 PMC11703871

[B18] Luo S. Yu J. Tong R. (2025). Research on accelerated geospatial computing methods for species distribution prediction-a case study of cold-water corals. J. Guizhou Univ. (Nat. Sci.) 42, 34–41. doi: 10.15958/j.cnki.gdxbzrb.2025.01.06

[B19] Montoya-Jimenez J. C. Valdez-Lazalde J. R. Ángeles-Perez G. De Los Santos-Posadas H. M. Cruz-Cárdenas G. (2022). Predictive capacity of nine algorithms and an ensemble model to determine the geographic distribution of tree species. iForest - Biogeosc. For. 15, 363–371. doi: 10.3832/ifor4084-015

[B20] Peters R. L. Darling J. D. S. (1985). The greenhouse effect and nature reserves: global warming would diminish biological diversity by causing extinctions among reserve species. BioScience 35, 707–717. doi: 10.2307/1310052

[B21] Rocchini D. Andreo V. Förster M. Garzon-Lopez C. X. Gutierrez A. P. Gillespie T. W. . (2015). Potential of remote sensing to predict species invasions: a modelling perspective. Prog. Phys. Geogr. 39, 283–309. doi: 10.1177/0309133315574659

[B22] Shu J. Peng X. Zhang X. Chen X. (2023). Prediction of Tibet's potential suitable areas for Picea brachytyla based on MaxEnt model. J. Northwest. For. Univ. 38, 66–72.

[B23] Song X. Cao M. Li J. Kitching R. L. Nakamura A. Laidlaw M. J. . (2021). Different environmental factors drive tree species diversity along elevation gradients in three climatic zones in Yunnan, southern China. Plant Divers. 43, 433–443. doi: 10.1016/j.pld.2021.04.006 35024512 PMC8720829

[B24] Su J. Piao Y. Luo Z. Yan B. (2018). Mapping potential distribution of wild bird using convolutional neural network. Comput. Syst. Appl. 27, 248–254. doi: 10.15888/j.cnki.csa.006571

[B25] Tang L. (2013). Diagnosis and control methods for major pests and diseases of Coptis. Contemp. Hortic. (11), 103–104. doi: 10.14051/j.cnki.xdyy.2013.11.025

[B26] Virgili A. (2018). Modelling distributions of rare marine species: the deep-diving cetaceans (La Rochelle, France: Université de La Rochelle). PhD Thesis.

[B27] Wang X. Jiang C. Xu G. (2011). Potential distribution of an invasive pest,Eriosoma lanigerum in China. Chin. J. Appl. Entomol. 48, 379–391.

[B28] Wang C. Liu C. Wan J. Zhang Z. (2016). Climate change may threaten habitat suitability of threatened plant species within Chinese nature reserves. PeerJ 4, e2091. doi: 10.7717/peerj.2091 27326373 PMC4911960

[B29] Wang J. Mu Q. Liu J. Shi X. (2013). Characteristics of Coptis teetoides community in Gaoligong Mountain. J. Anhui Agric. Sci. 41, 739–744, 747. doi: 10.13989/j.cnki.0517-6611.2013.02.132

[B30] Wang J. Ni J. (2006). Review of modelling the distribution of plant species. Chin. J. Plant Ecol. 30, 1040–1053. doi: 10.17521/cjpe.2006.0133

[B31] Wang W. Tang W. Gong Y. Wang X. Li Y. (2023). Modeling habitat of skipjack tuna of free swimming school in Western and Central Pacific Ocean based on MaxEnt model. South. China Fish. Sci. 19, 11–21.

[B32] Wang H. Zhou H. He J.-S. Lu C. Huang Y. Zhang J. . (2025). Divergent phenological responses of soil microorganisms and plants to climate warming. Nat. Geosci. 18, 753–760. doi: 10.1038/s41561-025-01738-9 37880705

[B33] Xi C. Mu L. Li S. Yue Y. He S. Gui F. . (2018). MaxEnt modeling and ArcGIS for predicting the potential distribution of Pistia stratiotes L. in Yunnan Province. J. Yunnan Agric. Univ. (Nat. Sci.) 33, 7–16.

[B34] Xia W. Chen X. Jiang Y. (2020). Research on academic paper citation prediction. Lib. Inf. Service 64, 138–145. doi: 10.13266/j.issn.0252-3116.2020.06.016

[B35] Xu Y. Xie D. Dong S. Ning Z. (2025). Quantitative characteristics of Coptis chinensis var. brevisepala populations in Guangdong Province and prediction of its potential distribution area in China based on MaxEnt. Chin. J. Ecol. 44, 3044–3053. doi: 10.13292/j.1000-4890.202509.022

[B36] Yang W. Jin H. Li W. Zhang Z. Zhao Z. Zhang J. (2013). A study on phenotypic diversity in different populations of endangered Coptis teeta Wall of Yunnan. J. Yunnan Univ. (Nat. Sci. Edition) 35, 719–726.

[B37] Yang Y. Xie S. Meng Z. Chen G. Liang Y. (2012). Pollination ecology of Coptis teeta Wall. an endangered medicinal plant. Acta Botanica Boreali-Occidentalia Sin. 32, 1372–1376.

[B38] Zhang X. (2018). Analysis on key techniques of Coptis chinensis cultivation. J. Green Sci. Technol. (9), 74–75, 77. doi: 10.16663/j.cnki.lskj.2018.09.033

[B39] Zhang H. Sun P. Zou H. Ji X. Wang Z. Liu Z. (2024). Adaptive distribution and vulnerability assessment of endangered maple species on the Tibetan plateau. Forests 15, 491. doi: 10.3390/f15030491 30654563

[B40] Zhao Q. Zhang Y. Li W.-N. Hu B.-W. Zou J.-B. Wang S.-Q. . (2021). Predicting the potential distribution of perennial plant Coptis chinensis Franch. in China under multiple climate change scenarios. Forests 12, 1464. doi: 10.3390/f12111464 30654563

